# A Strategy to Elicit M2e-Specific Antibodies Using a Recombinant H7N9 Live Attenuated Influenza Vaccine Expressing Multiple M2e Tandem Repeats

**DOI:** 10.3390/biomedicines9020133

**Published:** 2021-02-01

**Authors:** Daria Mezhenskaya, Irina Isakova-Sivak, Tatiana Kotomina, Victoria Matyushenko, Min-Chul Kim, Noopur Bhatnagar, Ki-Hye Kim, Sang-Moo Kang, Larisa Rudenko

**Affiliations:** 1 Department of Virology, Institute of Experimental Medicine, 197376 Saint Petersburg, Russia; dasmez@iemspb.ru (D.M.); kotomina@iemspb.ru (T.K.); matyshenko@iemspb.ru (V.M.); vaccine@mail.ru (L.R.); 2 Center for Inflammation, Immunity and Infection, Institute for Biomedical Sciences, Georgia State University, Atlanta, GA 30303, USA; mckim001@gmail.com (M.-C.K.); nbhatnagar1@student.gsu.edu (N.B.); kkim39@gsu.edu (K.-H.K.); skang24@gsu.edu (S.-M.K.)

**Keywords:** influenza, universal influenza vaccine, live attenuated influenza vaccine, M2e antigen, recombinant influenza virus, cross-protection, mouse model, CDC, ADCC, IgG

## Abstract

Influenza viruses remain a serious public health problem. Vaccination is the most effective way to prevent the disease; however, seasonal influenza vaccines demonstrate low or no effectiveness against antigenically drifted and newly emerged influenza viruses. Different strategies of eliciting immune responses against conserved parts of various influenza virus proteins are being developed worldwide. We constructed a universal live attenuated influenza vaccine (LAIV) candidate with enhanced breadth of protection by modifying H7N9 LAIV by incorporating four epitopes of M2 protein extracellular part into its hemagglutinin molecule. The new recombinant H7N9+4M2e vaccine induced anti-M2e antibody responses and demonstrated increased protection against heterosubtypic challenge viruses in direct and serum passive protection studies, compared to the classical H7N9 LAIV. The results of our study suggest that the H7N9+4M2e warrants further investigation in pre-clinical and phase 1 clinical trials.

## 1. Introduction

Despite the ongoing COVID-19 pandemic, influenza remains a serious public health concern, especially in light of a number of documented cases of SARS-CoV-2 and influenza A co-infections in humans [1,2,3,4]. Before the pandemic, the World Health Organization (WHO) estimated that seasonal influenza can cause up to 645,832 deaths around the world annually [5]. In addition, new antigenic variants of influenza viruses periodically arise in circulation causing devastating pandemics [6]. Vaccination is considered the first line of defense against influenza, but the ever-changing nature of influenza viruses result in ineffectiveness of vaccination after a single season or against new pandemic strains. Therefore, there is a need for more broadly protective influenza vaccines capable of protecting against all or diverse influenza viruses and induce long-lasting immunity.

Many different strategies aimed at improving the breadth and longevity of immune responses have been explored to address this issue with highly promising vaccines in pre-clinical and clinical evaluations [7,8]. Among several conserved influenza A viral proteins and epitopes, the ectodomain of matrix 2 protein (M2e) is a promising target for universal influenza vaccine design [9]. This domain is highly conserved among influenza A viruses isolated from various species; however, natural influenza infection is unable to induce significant immunity to the M2 protein [10] due to several reasons, which include its small size, small number of copies in the virion, and the possible shielding effect of larger surface proteins of influenza A virus [11,12,13].

Currently approved live attenuated influenza vaccines (LAIVs) are thought to stimulate mucosal and systemic IgA and IgG, as well as T-cell mediated immunity, nonetheless, similarly to natural virus infection, they are weak inducers of M2e-targeted antibody. We have recently developed a vaccine strategy that will result in enhanced M2e antibody responses after vaccinations by incorporation of four M2e tandem repeats into N-terminus of HA1 hemagglutinin subunit of seasonal LAIV strain [14]. A two-dose intranasal immunization of naïve BALB/c mice with the chimeric H1N1+4M2e or H3N2+4M2e elicited significant levels of M2e antibody which protected animals against heterologous influenza A virus challenge, as confirmed by serum passive transfer experiments. It is however likely that pre-existing immunity to seasonal influenza viruses in human population might interfere with replication of the LAIV viruses in the upper respiratory tract, thus potentially reducing the immunogenicity of the chimeric LAIV+4M2e vaccines [15,16]. Therefore, we further constructed the chimeric 4M2e-expressing LAIV based on a H7N9 influenza virus, which caused an outbreak in China in 2013 and since then has been sporadically detected in humans, but has not been circulated widely in the population, thus the population remains immunologically naïve [17,18]. Here, we conducted a comprehensive study of the H7N9+4M2e LAIV infectivity, safety, immunogenicity, and the ability to protect mice against antigenically diverged influenza variants with special attention to the immunological mechanisms of this enhanced cross-protection.

## 2. Materials and Methods

### 2.1. Plasmids, Viruses, Cells and Proteins

African green monkey kidney (Vero) cell line and Madin–Darby Canine Kidney (MDCK) cell line were obtained from ATCC (American Type Culture Collection, Manassas, VA, USA).

Vero cells were maintained in OptiPRO SFM media (Thermo Fisher Scientific, Waltham, MA, USA) supplemented with antibiotic-antimycotic and GlutaMAX (Thermo Fisher Scientific, USA) at 37 °C in the atmosphere of 5% CO_2_. MDCK cells were grown in DMEM media (Thermo Fisher Scientific, USA) with 10% fetal bovine serum (FBS) (Thermo Fisher Scientific, USA) and 1× antibiotic–antimycotic (Thermo Fisher Scientific, USA) at the same conditions as Vero cells.

For the development of universal vaccine prototype pCIPolISapIT plasmids encoding wild-type HA and NA genes of A/Anhui/1/2013 (H7N9) virus were used [19]. A plasmid encoding 4M2e tandem repeats (2 human, swine and avian origin) was chemically synthesized by GenScript (Piscataway, NJ, USA). The remaining six plasmids encoding internal and non-structural proteins of cold-adapted master donor virus A/Leningrad/134/17/57 (H2N2) were generated previously [20].

Influenza A viruses A/Puerto Rico/8/34 (H1N1), A/South Africa/3626/2013 (H1N1), A/Nanchang/993/95 (H3N2), and A/Hong Kong/1073/99 (H9N2) were from the influenza virus repository of the Department of Virology, IEM. A mouse-adapted H1N1 A/California/7/2009 virus was provided by Smorodintsev Research Institute of Influenza, Saint Petersburg, Russia. Virulent for mice viruses A/Philippines/2/82 (H3N2) and a PR8-based reassortant A/Vietnam/1203/04-PR8 (rgH5N1) were from the Center for Inflammation, Immunity and Infection, Institute for Biomedical Sciences, Georgia State University.

A recombinant 3M2e protein [21] was kindly provided by Dr. A. Kazaks (Latvian Biomedical Research and Study Centre, Riga, Latvia).

### 2.2. Generation of Recombinant LAIV Virus

The 4M2e epitopes were introduced into HA molecule of A/Anhui/1/2013 (H7N9) virus by standard gene engineering approaches. First, a BsmBI restriction site was introduced between a signal peptide (SP) and HA1-subunit of the full-length HA gene of A/Anhui/1/2013 (H7N9) virus, and the modified gene was cloned into a dual-promoter plasmid [22]. Then, the 4M2e sequence was amplified using specific primers with extended sequences both containing BsmBI sites. Finally, after restriction and ligation procedures, a dual-promoter plasmid containing the chimeric H7N9+4M2e gene was generated.

Chimeric LAIV virus expressing four M2e epitopes within chimeric HA was generated by the means of reverse genetics. Vero cells were electroporated by the corresponding plasmid set using Neon Transfection System (Invitrogen, Carlsbad, CA, USA) according to the manufacturer’s instructions. Electroporated Vero cells were incubated at 37 °C and 5% CO_2_ for 6 h, followed by media change and further 3 days of incubation at 33 °C and 5% CO_2_ [22]. Then, the cells were scraped, resuspended in media, and used for inoculation of 10-day-old chicken embryos. The eggs were incubated at 33 °C for 2 days, and the presence of the recombinant virus (H7N9+4M2e) was confirmed by hemagglutination assay. Virus stock was clarified by low-speed centrifugation and the virus was stored at −70 °C in single-use aliquots.

Full-length sequence of the rescued virus was generated by Sanger sequencing using an automatic capillary sequencer ABIPrism 3130xl (Applied Biosystems, Waltham, MA, USA) and commercial BigDye Terminator Cycle Sequencing Kit. Genetic stability of the recombinant virus was studied after ten sequential passages in chicken embryos and the presence of the 4M2e insert was also confirmed by gene sequencing.

### 2.3. Assessment of M2e Expression by Recombinant Influenza Virus

Egg-grown H7N9 and H7N9+4M2e viruses were purified by high-speed centrifugation using Optima L-100 XP Ultracentrifuge (Beckman Coulter, Brea, CA, USA). First, the viruses were pelleted by centrifugation at 34,000× *g* for 2 h. Then, sucrose density gradient (60% and 30%) ultracentrifugation at 67,000× *g* for 1.5 h was performed to remove contaminant proteins and cell debris. The virus-containing interface was carefully collected, diluted in PBS, and pelleted by final centrifugation at 67,000× *g* for 1 h. The concentration of viral proteins was measured using the Micro BCA Protein Assay Kit (Thermo Fisher Scientific, USA).

Purified viruses were studied by Western blotting protocol. All samples were preliminary temperature denatured in the presence of β-mercaptoethanol and Laemmli buffer. Then, the samples were resolved by SDS-PAGE (12–15% Mini-PROTEAN, BioRad), followed by protein transfer to PVDF membrane by semi-dry method. Afterwards, the membrane was blocked in 3% milk in phosphate-buffered saline (PBS), followed by staining with 1 µg/mL 14C2 primary antibody, which binds M2e protein (Abcam, Cambridge, UK) and diluted 1:2000 goat anti-mouse IgG (Abcam, UK) as a secondary antibody. Both antibodies were diluted in blocking solution. The signal was visualized using 3,3′-Diaminobenzidine (DAB) HRP substrate staining (Vector Laboratories, Burlingame, CA, USA). A protein molecular weight marker Spectra Multicolor Broad Range Protein Ladder was run along the virus protein samples (Thermo Fisher Scientific, USA).

In addition, MDCK cells were used to assess the expression of M2e epitopes on the surface of infected cells using cell ELISA. MDCK cells were infected with 10-fold dilutions of H7N9+M2e or H7N9, starting with 3 MOI (number of TCID_50_ per cell). After 24 h of incubation, plates were fixed with cold 80% acetone and incubated on ice for 20 min. Then, the plates were washed twice with PBS supplemented with 3% tween (PBST) and blocked with 50 μL of 5% non-fat dry milk in PBS at 37 °C. After 1h incubation, plates were washed with PBST and quenched with 50 μL of 0.8 M H_2_O_2_ at room temperature for 15 min. After an additional wash, primary 14C2 antibody (1 µg/mL) diluted in 5% non-fat dry milk in PBS were added and plates were incubated at 37 °C for 1 h, followed by washing with PBST and addition of diluted 1:3000 secondary anti-mouse IgG antibody (Abcam, UK). Afterwards, plates were washed 3 times, and antibody binding was detected with 1-Step Ultra TMB-ELISA Substrate Solution (Thermo Fisher Scientific, USA). Once the desired color developed (approximately 15 min), 50 μL of H_2_SO_4_ stop solution were added to each well. The absorbance was measured at 450 nm using xMark Microplate Spectrophotometer (BioRad, Hercules, CA, USA).

### 2.4. In Vitro Characterization of the H7N9+M2e Recombinant Influenza Virus

Temperature-sensitive (ts) and cold-adapted (ca) phenotypes of studied viruses were determined by end-point titration in chicken embryos incubated at different temperatures. The virus was considered “ts” if the titer at 38 °C was reduced ≥3.0 lgEID_50_ compared to 33 °C. The virus is considered “ca” if the titer at 26 °C is reduced by ≤3.0 lgEID_50_ compared to 33 °C. Virus titers in eggs were calculated using the Reed and Muench method [23] and expressed in log10 50% egg infectious doses (log_10_EID_50_/mL).

In addition, viral growth was assessed in MDCK cells to determine the 50% tissue culture infectious dose (TCID_50_) and viral growth kinetics. Growth of the influenza viruses on MDCK cells was determined using a standard protocol. Briefly, MDCK cells were seeded on 96-well plates the day before in DMEM (Gibco, Waltham, MA, USA) containing 5% FBS (Gibco, USA) and 1× antibiotic–antimycotic (Gibco, USA). On the day of experiments, the plates were washed twice with PBS and infected with 10-fold dilutions of H7N9+M2e or H7N9 virus, 6 wells per dilution in a total volume of 25 μL. Control wells were left uninfected. Viral culture medium (DMEM supplemented with 1× antibiotic–antimycotic and 1 µg/mL of TPCK-treated trypsin) was used as a viral diluent. The plates were incubated at 33 °C in a 5% CO_2_ atmosphere for 1 h. Then, the inoculum was removed and 150 μL of viral culture medium were added to each well. The plates were incubated at 33 °C in the atmosphere of 5% CO_2_ for 3 days. Viral titers were calculated using the Reed and Muench method [23] and expressed in log_10_ TCID_50_/mL.

To assess the kinetics of virus growth, MDCK cells were seeded on 6-well plates and the next day were infected with H7N9+M2e or H7N9 at a MOI of 0.01 TCID_50_ per cell using the protocol described above. Plates were incubated at 33 °C in a 5% CO_2_ atmosphere and supernatants were harvested every 12 h for 3 days and stored at −70 °C until analyzed by TCID_50_ assay as described above.

### 2.5. Replication of the H7N9+M2e Recombinant Influenza Virus in the Respiratory Tract of BALB/c Mice

To determine the 50% mouse infectious dose (MID_50_), BALB/c mice (*n* = 5) were inoculated with various doses of the recombinant LAIV+4M2e or control H7N9 LAIV virus (3 to 7 lgEID_50_). The LAIVs were administrated intranasally without adjuvant in a volume of 50 μL. Nasal turbinates and lungs were collected at Day 3 post-infection (dpi) and stored frozen at −70 °C until used for titration. Tissue homogenates were prepared using a small bead mill (TissueLyser LT, QIAGEN, Germany) in 1 mL of sterile PBS. Eggs were inoculated with 200 µL of clarified supernatants, incubated at 33 °C for 48 h, and the presence of the virus was detected by hemagglutinating activity assay. The number of infected mice was counted at each dilution and MID_50_ was calculated by the Reed and Muench method [23]. In addition, 50% egg infective dose (EID_50_) was determined in homogenates of the nasal turbinates and lungs of mice infected with 6 and 7 log_10_EID_50_ of each virus.

### 2.6. Immunization and Challenge

**Figure 1:**
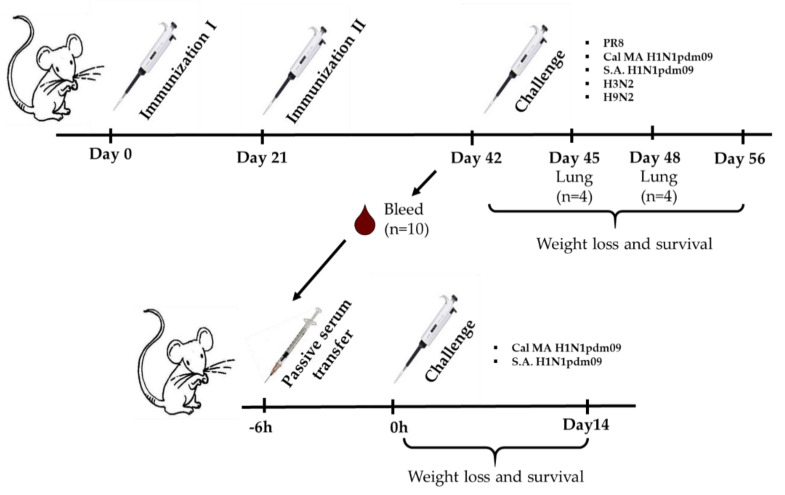
Overview of the mouse study design. BALB/c mice (*n* = 60) were immunized twice with a corresponding vaccine (H7N9, H7N9+4M2e, or PBS), three weeks apart. Serum samples were totally collected from 5 mice of each group on Day 42 for immunological assessment. On Day 45, the rest mice were challenged with heterosubtypic influenza viruses: 6 log_10_EID_50_ of A/Nanchang/993/95 (H3N2) and A/Hong Kong/1073/99 (H9N2); 3 LD_50_ of A/Puerto Rico/8/34 (PR8) and A/South Africa/3626/2013 (S.A. H1N1pdm09); and 30 or 300 LD_50_ of mouse-adapted A/California/7/2009 (Cal MA H1N1 pdm09). Protection was assessed by determining viral lung titers on Days 3 and 6 post challenge and/or by monitoring weight loss and survival for 14 days. In the case of S.A. H1N1pdm09, six days post challenge blood samples were collected for further immunological assessment. For serum passive transfer experiment, pooled immune sera were injected to naïve mice intravenously by retro-orbital injection 6 h prior to intranasal challenge with 1 LD_50_ of S.A. H1N1pdm09 or 3 LD_50_ of Cal MA H1N1pdm09 virus.

Immunogenic and protective properties of the recombinant H7N9+4M2e virus and the H7N9 control LAIV virus were assessed in BALB/c mice. Groups of 6–10 weeks old female BALB/c mice were immunized intranasally twice with the indicated viruses at a dose of 300 MID_50_ with a 3-week interval. The control group received a PBS solution (Figure 1). On Day 42 of the experiment, serum samples were collected for immunological analyses. In addition, pooled sera from this time point were used for passive immunization experiments. On Day 45, challenge viruses were administered intranasally in a total volume of 50 µL.

For assessment of direct protection, A/Nanchang/993/95 (H3N2), A/Hong Kong/1073/99 (H9N2), and A/South Africa/3626/2013 (S.A. H1N1pdm09) were administered at a dose 6 log_10_EID_50_; A/Puerto Rico/8/34 (PR8) was used at dose 4 log_10_EID_50_, which corresponded to 3 LD_50_. A mouse-adapted A/California/7/2009 (Cal MA H1N1 pdm09) virus was used at two doses, 4 and 5 log_10_EID_50_, which corresponded to 30 and 300 LD_50_, respectively. In the case of non-lethal A/Nanchang/993/95 (H3N2) and A/Hong Kong/1073/99 (H9N2) viruses, only lung viral titers of infected mice were studied on 3 and 6 d.p.i. In the case of lethal PR8, Cal MA H1N1 pdm09, S.A. H1N1pdm09 viruses, body weight loss and survival were monitored for 14 days after challenge.

For passive transfer, non-treated undiluted immune serum was administered to naïve BALB/c mice intravenously via retro-orbital injection (ROI) in a volume of 100 μL [24]. Six hours later, mice were challenged intranasally with indicated viruses.

For evaluation of protective properties of H7N9+4M2e and H7N9 vaccines after passive immunization, mice were infected with 3 log_10_EID_50_ of Cal MA H1N1 pdm09 and 5 log_10_EID_50_ of S.A. H1N1pdm09. Body weight loss and survival rate of infected mice were monitored daily for two weeks.

### 2.7. Assessment of Antibody Immune Responses

Serum samples were collected from the groups of immunized mice three weeks after the second dose. IgG antibody immune response, and IgG1/IgG2a subtypes were assessed by enzyme-linked immunosorbent assay (ELISA). High-sorbent 96-well plates (Corning, Glendale, AZ,, USA) were coated with 50 ng/well of either H7N9 sucrose-purified whole virus or recombinant 3M2e protein in a carbonate-bicarbonate buffer, in a volume of 50 µL per well at 4 °C overnight. Twofold dilutions of sera were prepared starting from 1:20 (for M2e antigen) or 1:100 (for H7N9 antigen) and added to the coated wells, which were then incubated with anti-mouse IgG, IgG1, or IgG2a conjugated to horseradish peroxidase (all from Sigma, USA). Antibody binding was detected with 1-Step Ultra TMB-ELISA Substrate Solution after incubation for 15 min at room temperature (Thermo Fisher Scientific, USA). Optical density was measured at 450 nm using xMark Microplate Spectrophotometer (BioRad, USA). The area under the curve (AUC) of the OD_450_ values for all sera dilutions was calculated using the trapezoidal rule and expressed in arbitrary units.

Functional activity of the induced antibodies was assessed by antibody-dependent natural killer (NK) cell degranulation assay, as a biomarker for antibody-dependent cellular cytotoxicity (ADCC) response [25]. Briefly, high-sorbent 96-well plates (Corning, USA) were coated with 50 ng/well of recombinant 3M2e protein in carbonate-bicarbonate buffer at 4 °C overnight. The next day, 5 µL of serum samples were diluted 10-fold in PBS and added to the coated wells, followed by incubation at 37 °C and 5% CO2 for 1 h. Then, 100 µL of CR-0 containing 3 × 10^6^ murine splenocytes collected from naïve C57BL/6J mice were added to each well and incubated at 37 °C and 5% CO_2_ for 24 h. Then, supernatants were collected and stained with ZombieAqua fixable viability dye, anti-CD3 (clone 2E7), anti-CD49b (clone DX5), anti-CD45.2 (clone 104), anti-CD107a (clone 1D4B) antibody-conjugates (Biolegend, San Diego, CA, USA) diluted in staining buffer (SB) (PBS supplemented with 0.2% BSA and 0.05% sodium azide), for 20 min in the dark place, followed by washing with 200 µL SB twice and resuspension of the cells in 1% formaldehyde. The cytolytic granule membrane protein CD107a was used to measure its mobilization to the cell surface, which occurs as the granule membrane merges with the cell membrane during degranulation [26]. Plates were stored in a dark cool place prior to flow cytometric analysis. At least 100,000 events were measured using a Navios flow cytometer (Beckman Coulter, USA). Data were analyzed using FlowJo software (TriStar Inc). The gating strategy is shown in Figure S1.

Functional activity of the induced antibody was also assessed by complement-dependent cytotoxicity assay. Monolayers of MDCK cells seeded the day before on 24-well plates were infected with a heterologous A/California/7/2009 (H1N1) influenza virus at 10 MOI and incubated in DMEM supplemented with antibiotic-antimycotic and 1 µg/mL TPCK trypsin at 37 °C in 5% CO2 overnight. After 18 h incubation, each well was tested for the presence of hemagglutinating activity to confirm active virus replication. Then, the medium was removed and cells were washed with 300 µL PBS, followed by addition of 50 µL of mouse serum diluted in DMEM 1:2 and incubation at 37 °C and 5% CO_2_ for 15 min. Then, 50 µL of guinea pig naïve sera diluted 1:10 in DMEM were added as a complement source and plates were further incubated for 3 h at 37 °C and 5% CO_2_. The supernatants were collected to the 2.0 mL tubes, and the remaining cell monolayers in the wells were washed twice with PBS, the cells were then dissociated by addition of Accutase and incubation for 10 min at 37 °C and 5% CO2. The Accutase was inactivated by addition of 1 mL DMEM supplemented with 10% FBS. The cells were thoroughly resuspended and collected to the same tubes with corresponding supernatants. This protocol ensures that all cells at different stages of apoptosis are collected from each sample. Tubes were centrifuged for 7 min, 300× *g*, washed with PBS twice, and resuspended in 300 µL PBS. Propidium iodide and YO-PRO iodide (Thermo Fisher Scientific, USA) were added to the tubes and the cells were immediately processed using a Navios Flow Cytometer. The percent of late apoptotic cells of cells in their late apoptotic phase was a measure of CDC antibody activity.

### 2.8. Statistical Analysis

Data were analyzed with the statistical module of Graph Pad Prism 6 software. The statistical significance of the difference between two groups (evaluation of viral titers in vitro and in vivo) was determined by the Mann–Whitney U-test. Differences between three groups (H7N9+4M2e, H7N9 and PBS) in ELISA and challenge assays were determined by ANOVA with Tukey’s multiple comparison test. Differences in the survival rates after challenge were analyzed by a log-rank Mantel–Cox test. *p* values of <0.05 were considered significant.

### 2.9. Ethic Statement

All mice experiments were approved by the Local Ethics Committee of the Institute of Experimental Medicine, Saint Petersburg (Approval No. 1/20 from 27.02.2020). Six- to eight-week-old female BALB/c mice were provided by Stolbovaya animal breeding nursery laboratory (Moscow region, Russia). For all intranasal procedures (immunizations and challenge), retro-orbital bleeding and retro-orbital injections mice were anesthetized with isoflurane. All efforts were made to minimize mice suffering.

## 3. Results

### 3.1. Generation and In Vitro Characterization of H7N9+4M2e LAIV Recombinant Virus

**Figure 2:**
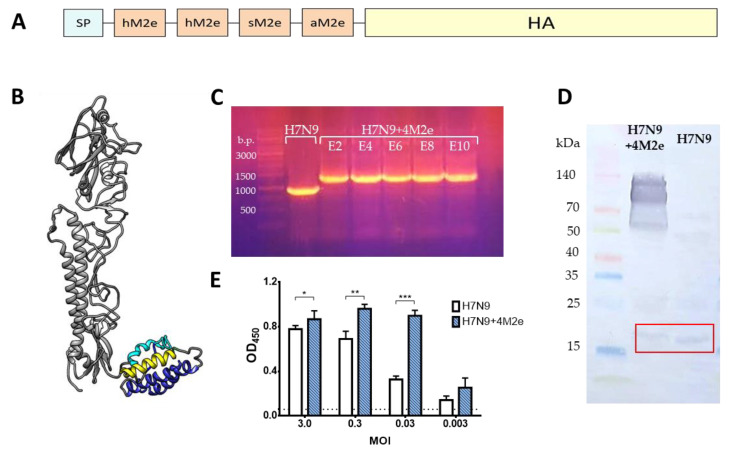
Generation of recombinant H7N9+4M2e virus and assessment of M2e expression. (**A**) Illustration of chimeric HA molecule with additional 4M2e epitopes inserted between the signal peptide and HA1 subunit (h, human; s, swine, a; avian). (**B**) Prediction of the 3D structure of the HA+4M2e monomer using M2e model after I-TASSER prediction. Human M2e epitopes are colored in blue, swine M2e is colored in yellow, avian M2e is colored in cyan. (**C**) Genetic stability of the 4M2e insertion within chimeric HA molecule after 10 sequential passages in eggs. (**D**) Western blot analysis of sucrose-gradient purified H7N9+4M2e and H7N9 LAIV viruses with M2e-specific 14C2 detection antibody. (**E**) Expression of M2e protein in MDCK cells infected with H7N9 and H7N9+4M2e at different MOIs as detected by binding with 14C2 antibody Day 1 post infection. Statistical significance between test groups was assessed by one-way ANOVA followed by a Tukey’s multiple comparisons test (* *p* < 0.05; ** *p* < 0.01; *** *p* < 0.001).

Recombinant LAIV virus expressing four M2e epitopes within chimeric HAs – H7N9 + 4M2e (Figure 2A) was rescued by the means of reverse genetics using plasmids encoding chimeric HA and intact NA genes of the A/Anhui/1/2013 (H7N9) and the six remaining genes of A/Leningrad/134/17/57 (H2N2) master donor virus. The identity of viral genes was confirmed by full-genome sequencing. Furthermore, the foreign 4M2e insert was genetically stable up to ten sequential passages in eggs (Figure 2C). In addition, we showed that the H7N9+4M2e recombinant virus was genetically stable, i.e., the insert was preserved after serial passages of the viruses in eggs and no mutations have been detected in the viral genome by passage E10. Western blot analysis of sucrose gradient-purified H7N9+4M2e and control H7N9 viruses revealed binding of M2e-specific 14C2 monoclonal antibody with recombinant H7N9+4M2e virus proteins with molecular weights approximately 55, 100 and 130 kDa, which most likely correspond to the HA1 subunit, HA0 protein and an oligomeric form of the HA [27] (Figure 2D). In contrast, the classical counterpart H7N9 LAIV did not show these bands, whereas binding with this antibody was seen for the M2 proteins of both viruses (molecular weight ~15 kDa), consistent with low levels of M2 proteins in virus.

To date, the 3D structure of free M2e has not been resolved. However, structure prediction based on amino acid sequence [28] suggests that 4M2e epitopes form compactly folded structure. The flexible linker between HA-subunit and 4M2e allows additional epitopes to be at a sufficient distance from the HA-stem and not to disturb its replication properties (Figure 2B). Despite the lack of information about M2 protein structure, the proposed structure of 4M2e-epitopes may represent native-like conformation reactive to M2e antibodies (Figure 2E).

Furthermore, the expression of excess M2e proteins on the surface of H7N9+4M2e-infected cells, compared to the H7N9-infected cells, was confirmed by cell ELISA. Thus, the 14C2 antibody binding was significantly increased when the cells were infected with the recombinant virus, compared to the cells inoculated with the same dose of the control LAIV strain (Figure 2E). It is known that the M2e protein is abundantly expressed by influenza viruses within an infected cell, but only a few copies are incorporated into virion [12,13]. Therefore, significant 14C2 binding was seen for the H7N9-infected cells at the higher MOIs, but this signal decayed rapidly with virus dilutions. In contrast, significant 14C2 binding of H7N9+4M2e-infected cells was observed even at low MOIs, suggesting that the M2e-specific antibody binds not only to the membrane-anchored M2 protein but also to the M2e epitopes expressed within the chimeric HA+4M2e conjugate.

**Figure 3:**
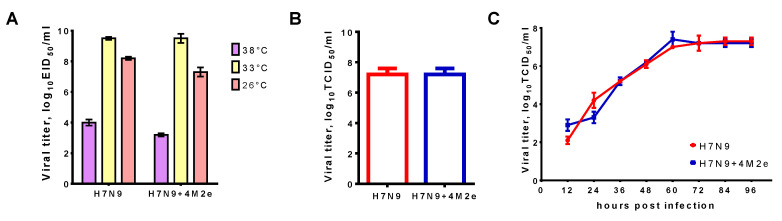
Growth characteristics of H7 and H7N9+4M2e viruses in vitro. (**A**) Replicative activity of influenza viruses at different temperatures in chicken embryos. (**B**) End-point titers of H7N9 and H7N9+4M2e viruses on MDCK cells at 33 °C. (**C**) Kinetics of LAIV viral growth in MDCK cells at 33 °C.

The ability of LAIV to replicate in susceptible cells is an indication for the ability of the vaccine to induce strong antibody and cell-mediated immune responses [29]. Comparison of replication level in eggs at 33 °C (Figure 3A) did not reveal any significant difference between classical LAIV (H7N9) and recombinant LAIV+M2e (H7N9+4M2e). Furthermore, both LAIVs shared identical ts/ca-phenotypes, suggesting that replacement of classical HA in LAIV strain by the modified H7+4M2e had no effect on virus replicative properties. Of note, maintenance of the *ts* phenotype is important for the LAIV strain, since such vaccine will replicate only in the upper respiratory tract of the vaccinated, inducing specific immunity in the “entrance gate” of preventing the infection, but it will not replicate in the lower respiratory tract, i.e., will not cause clinical signs of influenza infection.

Current influenza vaccines are produced using egg-based technology; however, cell culture-based technology might improve the safety of the vaccine, especially for those who have contraindications to vaccination due to the presence of allergic reactions to chicken proteins. In addition, in the event of an avian influenza virus pandemic, the new technology will allow the vaccine to be produced in large volumes under a risk of losing the main substrate. Therefore, we assessed the end-point titers and the kinetics of the H7N9+4M2e virus on MDCK cells, a most widely used substrate for influenza vaccine culture-based production. Comparison of H7N9+4M2e chimeric virus with H7N9 classical counterpart revealed the absence of any negative effect of the 4M2e insert on the replicative characteristics of the H7N9 LAIV virus (Figure 3B,C). These results suggest that the insertion of 4M2e epitopes into the HA molecule did not interfere with HA functional activity or alter major LAIV properties.

### 3.2. In Vivo Characterization of H7N9+4M2e LAIV Recombinant Virus

The ability of the new H7N9+4M2e vaccine candidate to replicate in the respiratory tract was studied in BALB/c mouse model. Mice were infected intranasally with 10^3^ to 10^7^ EID_50_ of the recombinant virus and the control LAIV strain and viral growth in nasal turbinates and lungs was assessed three days after inoculation. Virus detection at different infectious doses allowed calculating MID_50_ values which were comparable between the two groups. Importantly, the virus was more readily detected in NTs than in lungs, resulting in lower MID_50_ values (Table 1). Furthermore, virus titration in the upper and lower respiratory tracts after inoculation with higher vaccine doses (7 lg and 6 lg) revealed higher titers in the NTs compared to the viral pulmonary titers, suggesting that the attenuated phenotype of the chimeric virus was maintained (Table 1).

Overall, in vitro and in vivo studies of the H7N9+4M2e vaccine candidate provided the evidence that the insertion of 4M2e into HA molecule of the classical LAIV strain did not affect viral replicative characteristics and phenotypical properties.

### 3.3. Immunogenicity of H7N9+4M2e LAIV Recombinant Virus in BALB/c Mice

**Figure 4:**
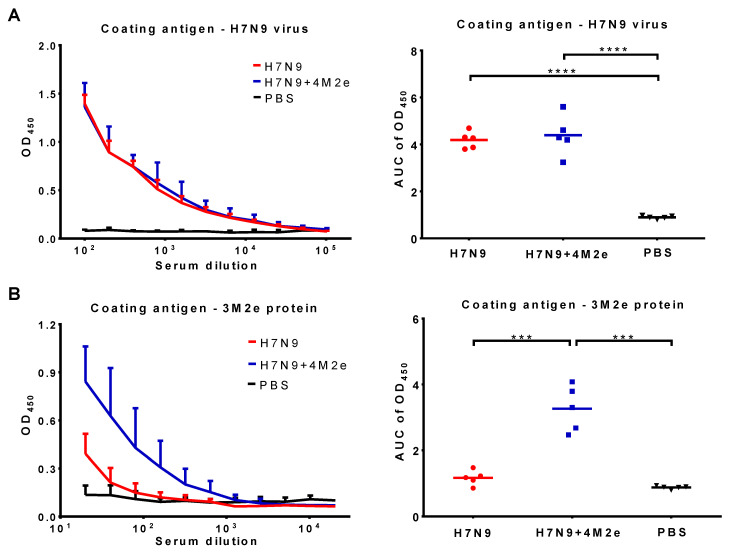
Serum IgG antibody responses in BALB/c mice immunized with H7N9+4M2e and H7N9 LAIVs. Serum samples were collected three weeks after the second vaccine dose and analyzed in ELISA using H7N9 whole virus (**A**) or 3M2e protein (**B**) antigens: (Left) the mean+SD OD_450_ values; and (Right) the area under the OD_450_ curve (AUC) values for individual animal. The AUC data were analyzed with one-way ANOVA followed by a Tukey’s multiple comparison test (*** *p* < 0.001; **** *p* < 0.0001).

**Figure 5:**
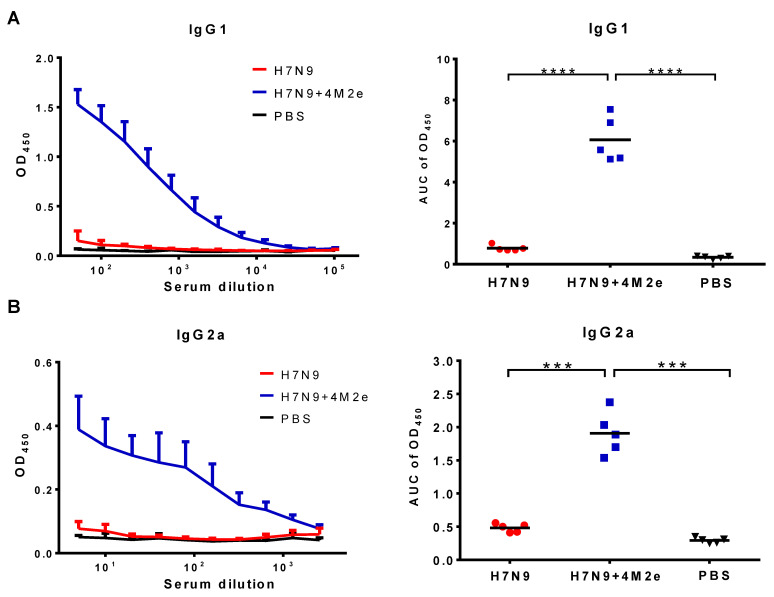
Serum IgG isotypes in BALB/c mice immunized with two doses of H7N9, H7N9+4M2e and PBS. IgG1 (**A**) and IgG2a (**B**) levels were assessed in ELISA: (Left) the mean+SD OD_450_ values; and (Right) the AUC values for individual animal. The AUC data were analyzed with one-way ANOVA followed by a Tukey’s multiple comparison (*** *p* < 0.001; **** *p* < 0.0001).

Groups of BALB/c mice were intranasally immunized with 300 MID_50_ of H7N9+4M2e vaccine and a classical control H7N9 LAIV virus, and three weeks later the mice were boosted with the same vaccine doses. Antibody immune responses to the vaccines were studied both prior to challenge (three weeks after the booster vaccine dose) and after a challenge with heterologous H1N1 virus. Primary immunogenicity outcome was the induction of serum IgG antibody responses analyzed in ELISA against a whole virus or recombinant 3M2e protein antigens. As shown in Figure 4, both vaccines induced similarly high levels of anti-influenza antibodies (Figure 4A). In contrast, only H7N9+4M2e vaccine could induce anti-M2e antibodies at significantly higher levels compared to H7N9 and PBS groups (Figure 4B). Furthermore, we studied the IgG1/IgG2a subclass profile of the induced anti-M2e antibody. Both IgG1 and IgG2a M2e-binding antibodies were detected at high levels in the H7N9+4M2e vaccine group, with slightly higher proportion of IgG1 antibody, compared to IgG2a (Figure 5). Interestingly, the classical H7N9 LAIV could induce some levels of M2e-binding antibody, which were further boosted by heterologous H1N1 influenza virus challenge, although these levels remained significantly lower than that of the H7N9+4M2e vaccine group (Figure S2).

### 3.4. Active Vaccination and Protection against Heterologous Influenza Viruses

**Figure 6:**
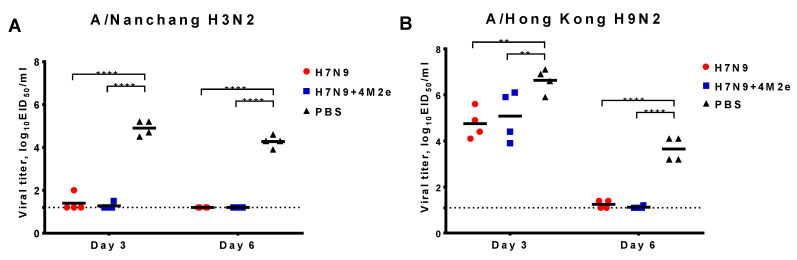
Protection of immunized mice against non-lethal viruses. Mice were immunized with two doses of H7N9, H7N9+4M2e, or PBS and challenged with 6lg EID_50_ of either A/Nanchang/993/95 (H3N2) (**A**) or A/Hong Kong/1073/99 (H9N2) (**B**). Viral pulmonary titers were assessed on Days 3 and 6 post challenge. Data were analyzed with two-way ANOVA followed by a Sidak’s multiple comparison test (** *p* < 0.01; **** *p* < 0.0001).

To find out whether the induction of M2e-specific antibodies by the recombinant H7N9+4M2e vaccine can enhance the cross-protective efficacy of H7N9 LAIV, we challenged immunized mice with a panel of divergent influenza A viruses. The H3N2 and H9N2 challenge viruses were not lethal for BALB/c mice; therefore, the protection was assessed by virological endpoints, i.e., by the reduction of virus pulmonary titers compared to the control mock-immunized animals. In the case of A/Nanchang/993/95 (H3N2) challenge, we observed full protection—the virus was almost cleared from the lungs of vaccinated mice at Day 3 post challenge, while the control mice shed the virus at significant levels (Figure 6A). No differences were observed between the vaccine groups, suggesting that the protection was mainly driven by the LAIV vaccine properties as H3 and H7 hemagglutinins belong to the same HA Group 2. Strikingly, challenge with H9N2 heterologous virus, which belongs to HA Group 1, also did not reveal significant differences between the vaccine groups: both LAIVs were equally protective, reducing virus titers at Day 3 and clearing the virus by Day 6 after challenge (Figure 6B). These data emphasize the cross-protective nature of the LAIV at least in mice, which might be attributed to the induction of cross-reactive T-cell responses.

**Figure 7:**
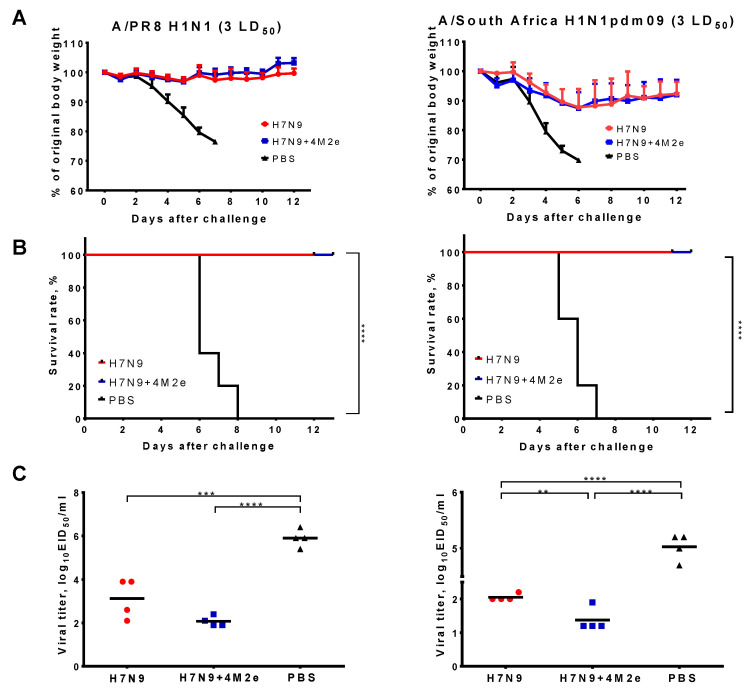
Protection of immunized mice against low doses of lethal viruses. Mice were immunized with two doses of H7N9, H7N9+4M2e, or PBS and challenged with 3 LD_50_ of either A/PR/8/34 (H1N1) (left) or A/South Africa/3626/2013 (H1N1pdm09) (right). Body weight loss (**A**) and survival rates (**B**) were monitored for two weeks post-challenge. Viral pulmonary titers (**C**) were assessed on Day 6 post challenge. Survival rates were compared by Mantel–Cox log-rank test. Virological data were analyzed with one-way ANOVA followed by a Tukey’s multiple comparison test (** *p* < 0.01; *** *p* < 0.001; **** *p* < 0.0001).

Challenge of immunized mice with heterologous PR8 H1N1 and SA H1N1pdm09 viruses at dose 3 LD_50_ showed similar protection to both H7N9 and H7N9+4M2e vaccines, as demonstrated by the absence of significant weight loss and 100% survival rates, while 100% of the mock-immunized animals succumbed to the infection by Day 7 post challenge (Figure 7A,B). Virological endpoints though revealed slightly better protection of the H7N9+4M2e vaccine, compared to the classical LAIV counterpart: on Day 6 after challenge in the H7N9+4M2e vaccine group, the virus almost cleared from the lungs, whereas some animals from the LAIV control group still shed the virus at a significant level (Figure 7C). Although the difference between the vaccine groups was not significant for the PR8 challenge (*p* = 0.07), significant difference in the SA H1N1pdm09-challenged animals suggests better protection afforded by the 4M2e-containing LAIV compared to the classical LAIV strain.

**Figure 8:**
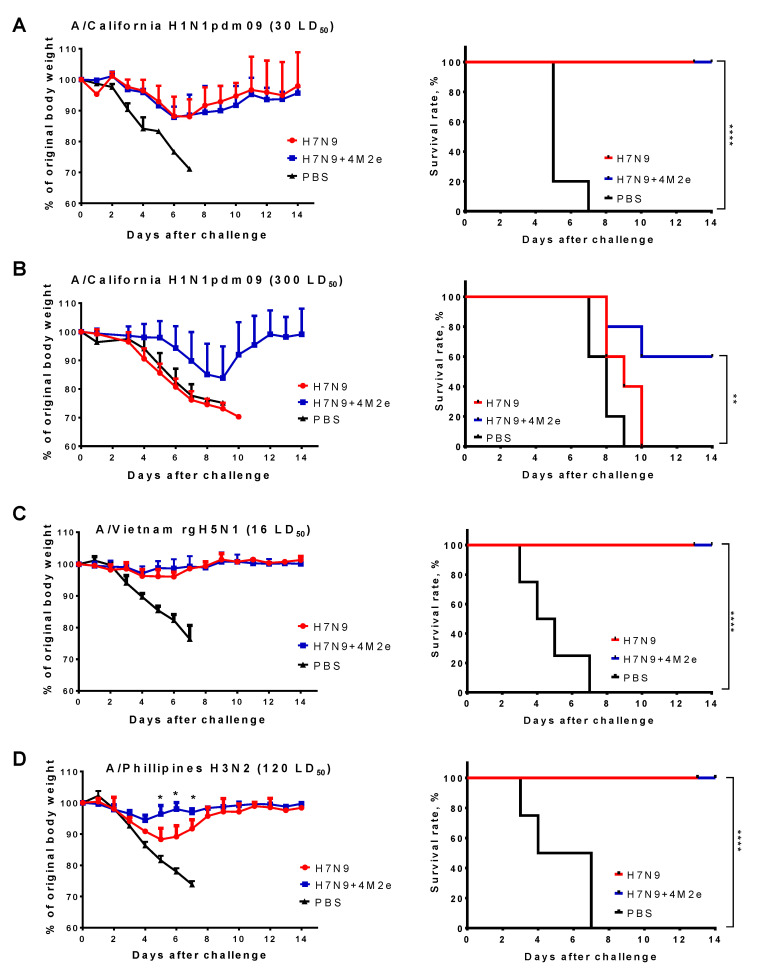
Protection of immunized mice against medium and high doses of a lethal heterologous virus. Mice were immunized with two doses of H7N9, H7N9+4M2e, or PBS and challenged with either 30 LD_50_ (**A**) or 300 LD_50_ (**B**) of a mouse-adapted A/California/7/09 (H1N1pdm09) virus; 16 LD_50_ of A/Vietnam/1203/04-PR8 (rgH5N1) (**C**) and 120 LD_50_ of A/Philippines/2/82 (H3N2) (**D**). Body weight loss (left panel) and survival rates (right panel) were monitored for two weeks post-challenge. Body weights were compared by Mann–Whitney test. Survival rates were compared by Mantel–Cox log-rank test. * *p* < 0.05; ** *p* < 0.01; **** *p* < 0.0001.

We also performed additional challenge experiments with a panel of virulent viruses in mice, such as mouse-adapted A/California/7/2009 (H1N1pdm), A/Philippines/2/82 (H3N2), and a PR8-based reassortant A/Vietnam/1203/04-PR8 (rgH5N1), to assess possible protective effect of the H7N9+4M2e-induced immunity against higher doses of a lethal heterologous virus. Challenge with 30 LD_50_ of A/California/7/2009 and 16 LD_50_ of rgH5N1 viruses again revealed similar protection, as observed in both vaccine groups (H7N9+4M2e and H7N9) compared to the PBS group: although immunized mice lost approximately 13% of the original body weight, all of them successfully recovered by Day 14, whereas mock-immunized mice did not survive this challenge (Figure 8A,C). Strikingly, increasing the dose of A/California/7/2009 challenge virus 10-fold (300 LD_50_) resulted in 100% lethality in the H7N9 LAIV group, whereas 60% of mice immunized with H7N9+4M2e survived the challenge (Figure 8B). Similarly, mice immunized with H7N9+4M2e vaccine were better protected against challenge with 120 LD_50_ of A/Philippines H3N2 virus, compared to the H7N9 LAIV group, which was evidenced by significantly reduced weight loss during the challenge phase (Figure 8D). These data suggest that M2e immunity might have played a role in protecting mice against a high dose of different heterologous influenza viruses belonging to Group 1 and 2 HAs.

Overall, the results of active immunization demonstrated a significant level of cross-protection afforded by classical H7N9 LAIV vaccine in mice, probably due to the action of cross-reactive T-cell based immunity. Nevertheless, the insertion of additional M2e epitopes into LAIV genome has advantages for conferring the protection against a high doses of lethal challenge viruses, suggesting the accessory protective role of the M2e-targeted immunity.

### 3.5. Passive Protection against Heterologous Influenza Viruses

**Figure 9:**
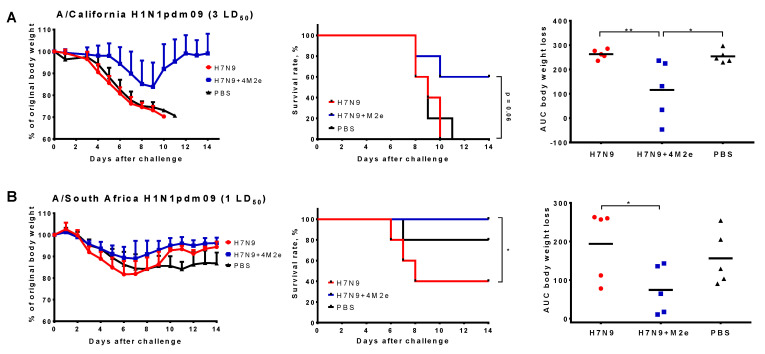
Protective effect of antibodies induced by study vaccines in a passive serum transfer experiment. Serum samples from mice immunized with two doses of H7N9, H7N9+4M2e, or PBS were pooled and injected intravenously to naïve BALB/c mice 6 h prior to challenge with either 3 LD_50_ of a mouse-adapted A/California/7/09 (H1N1pdm09) (**A**) or 1 LD_50_ of A/South Africa/3626/2013 (H1N1pdm09) virus (**B**). Body weight loss (left) and survival rates (middle) were monitored for two weeks post-challenge. Area under the curve of body weight loss (right) was an additional measure of protection. Survival rates were compared by Mantel–Cox log-rank test. AUC values were compared by one-way ANOVA, followed by Tukey’s multiple comparisons test. * *p* < 0.05; ** *p* < 0.01.

To study the impact of LAIV-induced antibodies on cross-protection without the interference of T cell immunity, we performed experiments on passive immunization of mice with immune sera, followed by infection with virulent heterologous viruses. Undiluted non-treated pooled sera from immunized mice were administered intravenously to naïve BALB/c mice and protective effect of antibodies was assessed by monitoring weight loss and survival rates for two weeks post-challenge. Challenge with two H1N1pdm09 viruses at doses 1 LD_50_ (for S.A.) and 3 LD_50_ (for MA Cal/09) revealed significant differences in the body weight change and/or survival rates between the H7N9 and H7N9+4M2e groups, suggesting positive impact of M2e-targeted antibodies on the cross-protection (Figure 9).

Overall, passive protection experiment revealed the improved protection afforded by LAIV containing additional M2e epitopes, most likely mediated by the M2e-specific antibodies. Since our immunogenicity assessment of the H7N9 and H7N9+4M2e LAIVs revealed no virus-neutralization effect of the immune sera against the H1N1 heterologous challenge viruses (data not shown), different immunological mechanisms are involved in the protective effect of the M2e-targeted antibodies.

### 3.6. Functional Activity of the M2e-Specific Antibody

Due to the non-neutralizing nature and the obvious impact of the M2e-specific antibody on the LAIV-induced cross-protective potential, it is important to assess functional activity of these antibodies, i.e., Fc-driven cytotoxicity effects of the antibody. Functional activity of the M2e-targeted antibody was assessed using two assays—complement dependent cytotoxicity and an antibody-dependent natural killer (NK) degranulation activity, which is a surrogate assay for antibody-dependent cellular cytotoxicity (ADCC) assay.

#### 3.6.1. Complement Dependent Cytotoxicity (CDC)

**Figure 10:**
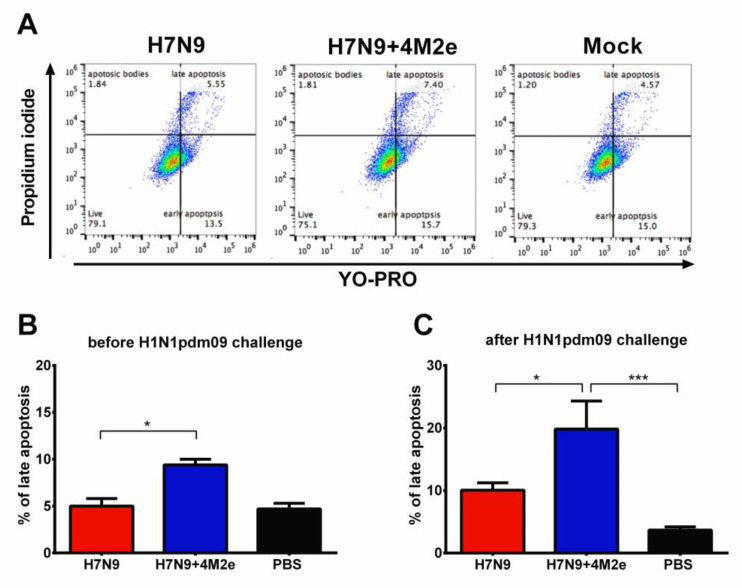
Functional activity of vaccine-induced antibodies as measured by complement-dependent cytotoxicity (CDC) assay. CDC activity was assessed by incubating mouse serum samples with H1N1 virus-infected MDCK cells in the presence of complement. (**A**) Representative plots of flow cytometric analysis of MDCK cells stained with YO-PRO and Propidium iodide. CDC activity of mouse serum samples was studied three weeks after the second immunization of H7N9, H7N9+4M2e, or PBS (**B**) or on Day 6 after challenge with SA H1N1 virus (**C**). Data were compared by one-way ANOVA, followed by Tukey’s multiple comparisons test. * *p* < 0.05; *** *p* < 0.001.

Mechanisms of protection afforded by M2e-based vaccines still raise some questions. One of the controversial points is whether C3 complement system plays important role in protection or not [30,31]. Serum samples from mice immunized with the study vaccines were added to MDCK cells infected with A/South Africa/3626/2013 (H1N1) virus, followed by incubation with complement (guinea pig naïve sera), which resulted in the infected cells entering different apoptosis phases. Here, the proportion of cells in their late apoptotic phase corresponded to the CDC antibody activity [32]. Interestingly, right after vaccination (Day 42 of the study), serum antibody from H7N9+4M2e-immunized mice demonstrated slightly higher CDC activity, compared to the H7N9 vector control group (Figure 10A,B), although the percent of late apoptotic cells was still much lower than in the positive control group (a hyperimmune mouse sera to the H1N1 virus). Importantly, sera collected from H7N9+4M2e immunized mice six days after challenge with S.A. H1N1 virus demonstrated significant increase in the CDC activity, and the percent of cells entering late apoptosis in the presence of these antibodies was significantly higher than that of H7N9- and mock-immunized mice (Figure 10C). Since the LAIV+4M2e-immunized mice had significantly reduced viral pulmonary titers six days post challenge compared to the vector-immunized group, the increase of CDC response in the chimeric vaccine group suggests that complement system plays an important role in reducing viral titers in the lungs, which is consistent with the previously published results of Wang et al. [31].

#### 3.6.2. Antibody-Dependent Cellular Cytotoxicity (ADCC)

**Figure 11:**
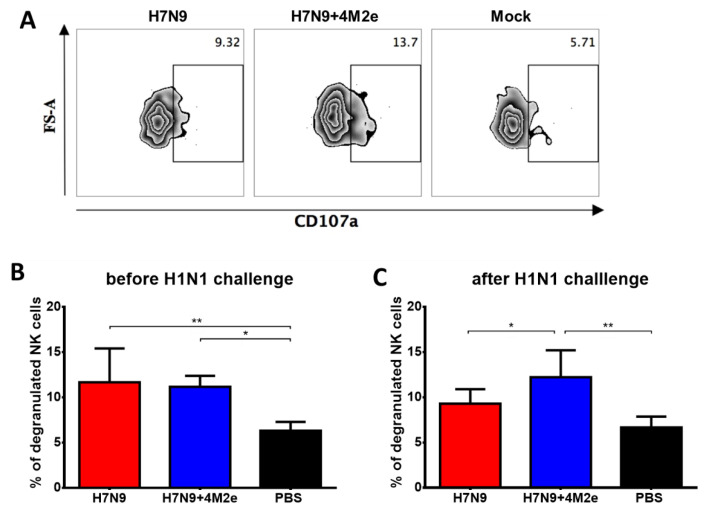
Functional activity of vaccine-induced antibodies as measured by antibody-dependent NK degranulation activity assay, a surrogate assay for ADCC. NK degranulation activity was assessed by incubating M2e-binding antibody present in mouse serum samples with splenocytes of naïve C57BL/6J mice as a source of NK cells. (**A**) Representative plots of flow cytometric analysis of NK cells degranulation. NK cell degranulation in the presence of mouse serum samples was studied three weeks after the second immunization of H7N9, H7N9+4M2e, or PBS (**B**) or on Day 6 after challenge with SA H1N1 virus (**C**). Data were compared by one-way ANOVA, followed by Tukey’s multiple comparisons test. * *p* < 0.05; ** *p* < 0.01.

Antibody-dependent cellular cytotoxicity of the induced antibody was similar in both vaccine groups on Day 42 of the study, as evidenced by the level of NK cell degranulation induced by incubation of the M2e-binding antibody with splenocytes of naïve C57BL/6J mice as a source of NK cells (Figure 11A,B). Although there was an increase of degranulation activity in the H7N9+4M2e group after S.A. (H1N1pdm09) challenge and the difference with H7N9 and PBS groups became statistically significant, this level of difference remained relatively low. This result suggests that the ADCC mechanism might not have a pivotal role in the protection, consistent with the study by Fu et al. of M2 monoclonal antibodies by other M2e-based vaccines [33].

## 4. Discussion

Despite the limited detection of influenza virus circulation in 2020 in the South Hemisphere due to the emergence of SARS-CoV-2 and robust anti-COVID-19 measures taken to contain the infection, public health authorities recommend that influenza vaccination campaigns are to be maintained and the vaccination coverage should reach as high a level as possible [34,35]. Influenza vaccines currently licensed for seasonal use have variable levels of effectiveness; possible reasons include frequent mismatches between the vaccine strain and circulating viruses, differences in vaccine formulations, adapting mutations arising during the process of vaccine production in eggs, immune memory from previous infections and vaccination, and possible immune imprinting effects [36,37]. The main mode of action of the licensed influenza vaccines is the induction of antibodies targeted to the viral surface antigens, hemagglutinin (HA) and neuraminidase (NA), mainly to their immunodominant hypervariable regions. Therefore, one of the possible solutions to increase vaccine effectiveness is to re-direct the immune response to some conservative viral antigens, such as HA stalk domain, the enzymatic site of NA, or extracellular part of M2 proteins [9,38]. Other strategies aim to induce cross-reactive T-cell immune responses to the conserved epitopes of the virus internal proteins, such as nucleoprotein and M1 [39,40]. There are approaches to enhance the immunogenicity of otherwise weak conserved viral epitopes, and viral vectors are among the promising platforms for this purpose due to their ability to induce both B- and T-cell responses to the inserted antigen of interest [41]. Here, we explored the possibility of licensed live attenuated influenza vaccine backbone as a viral vector to deliver additional conserved M2e epitopes of influenza A viruses to the target cells to enhance the breadth of protection afforded by classical LAIV viruses. It is well known that LAIVs induce cross-reactive immunity with a potential to protect against drifted influenza viruses in a variety of animal models as well as in human trials [15,42,43,44], and numerous studies have proven that LAIV is a promising viral vector for designing vaccines against other infectious pathogens [45,46,47,48]. Our recent study demonstrated that seasonal H1N1 and H3N2 LAIVs expressing four M2e tandem repeats within the chimeric HAs can elicit M2e-specific antibody and, to a lesser extent, T-cell responses which enhanced cross-protective potential of the vaccines [14]. With a view to the possible future clinical trials of LAIV+4M2e vaccines, it is important to minimize the interference of pre-existing immunity to seasonal influenza viruses with replication of the attenuated vaccine viruses in the upper respiratory tract of the volunteers; therefore, we developed a new M2e-based universal vaccine candidate based on pre-pandemic LAIV strain of H7N9 subtype. This zoonotic virus occasionally causes human infection, but the majority of the population remains immunologically naïve. Furthermore, the H7N9 LAIV has been previously shown to be safe and immunogenic in a ferret model [49] and in a phase 1 clinical trial [50]. Importantly, the H7N9 LAIV was the most immunogenic vaccine for healthy adults among all previously tested pre-pandemic LAIVs, suggesting its utility for the development of a universal influenza vaccine candidate.

The new H7N9+4M2e recombinant vaccine retained replicative properties comparable to the classical H7N9 LAIV both in vitro and in vivo. As expected, the H7N9+4M2e expressed much higher number of M2e chimeric protein copies within the infected MDCK cells, compared to the classical counterpart, and the additional M2e epitopes in the chimeric virus were identified along with the viral HA1 subunit in Western blot analysis, confirming the correct functional activity of the chimeric HA+4M2e proteins. The absence of negative effect of the 4M2e insert on LAIV properties was also confirmed by the induction of comparable immune responses against H7N9 LAIV backbone virus. The increased M2e-specific antibody immune responses by the H7N9+4M2e LAIV compared to the H7N9 LAIV indicate that this insert is correctly folded within the chimeric protein and structurally mimics the natural M2e epitopes.

The protective potential of the induced immune responses was assessed using a panel of heterosubtypic influenza A viruses. As was stated above, even classical LAIV had a significant level of protection against heterosubtypic challenge viruses administered at doses up to 30 LD_50_. However, the H7N9 LAIV showed no protection against a high-dose H1N1 challenge virus, whereas the recombinant H7N9+4M2e vaccine demonstrated significant protection of mice against lethality and body weight loss, compared to the H7N9 LAIV and mock-immunized animals. Serum passive transfer experiment confirmed that the enhanced protection induced by the chimeric vaccine is mediated by antibody immune responses, most probably by anti-M2e antibodies. According to our previous findings, the M2e-targeted antibody can be actively secreted by B-cells in mediastinal lymph nodes (MLN) post-challenge, suggesting the major mechanisms of immune protection afforded by LAIV+4M2e vaccines [14].

Despite decades of intensive studies, there are still no clearly established immune mechanisms of protection mediated by the M2e-based vaccines, although it is agreed that M2e-specific antibodies should play an important roles since the M2 protein is abundantly expressed in the infected cells [51]. The antibodies induced by our H7N9+M2e vaccine candidate were unable to neutralize heterosubtypic viruses used for the challenge, suggesting that the M2e-targeted antibodies do not possess neutralizing activity, which is in line with the results of other studies [33,52,53]. Therefore, other mechanisms are involved in the protective potential of this antibody subset, including their Fc-mediated functional activity. Thus, the observed high levels of anti-M2e IgG1 antibody could have played a crucial role in cross-protection since it was previously shown that functional interaction between these antibodies and FcγRIII receptor is essential for the protection in the mouse model [54].

We also observed significant induction of IgG2a antibodies by the recombinant vaccine, which have pro-inflammatory activity leading to the elimination of the virus-infected cells [55]. Although the induction of this IgG2a isotype was slightly lower than the IgG1 isotype, Van den Hoecke et al. showed that the M2e-specific IgG2a antibody protected better against influenza A virus challenge than the M2e-specific IgG1 antibody even at very low concentrations [56]. A possible explanation of this observation could be that IgG2a acts via FcγRI, which is the only known high-affinity receptor (10^8^–10^9^ M^−1^) in mice and humans and binds only IgG2a. All other receptors have 10–100 folds lower affinity and show a broader IgG subclass specificity [55].

Other functional activity of the induced antibody might play a role in protection, such as ADCC and CDC. The higher complement dependent cytotoxicity observed in H7N9+4M2e-immunized mice could have led to the faster viral clearance from the lungs and better recovery after H1N1 virus challenge, as this activity increased after challenge in the chimeric vaccine group. Despite the previously published data on importance of antibody-dependent cellular cytotoxicity mechanism in protection in mice [30,31,57], we did not observe significant differences in the ADCC activity between the H7N9 and H7N9+4M2e vaccines using an in vitro NK degranulation assay as a surrogate marker of ADCC. The level of induced anti-M2e IgG2a antibodies was probably not sufficient for this mechanism of protection, and this is in line with findings that in mice ADCC is primarily mediated by FcγRIV and is induced by IgG2a antibody isotype [58]. Nevertheless, further in vivo studies of ADCC antibody induced by LAIV+4M2e vaccines using a FcR knockout mouse model will help to find out whether the M2e-specific antibody possess this functional activity and if this mechanism contributes to the cross-protection.

Although we did not study T-cell immunity here, our previous findings observed for similar chimeric H1N1+4M2e and H3N2+4M2e vaccines can be extrapolated to the H7N9+4M2e vaccine as well, suggesting weak induction of M2e specific T cell immunity [14]. Similarly, weak M2e-specific CD4+ T cell responses were detected in mice immunized with VLP-based M2e vaccine, even though it induced much higher levels of M2e-specific antibody [21]. There are controversies about the contribution of the M2e-specific CD4+ and CD8+ T cell responses in the protection against heterologous influenza A viruses. Some studies suggest that both T-cell subsets play role in the cross-protection [59,60,61], whereas others demonstrated crucial role of M2e-specific CD4+, but not CD8+ T cells [62]. In contrast, Eliasson et al. showed that M2e-specific CD4+ T cells cannot convey sufficient protection against lethal challenge in the absence of M2e-specific antibodies [62].

Despite the high immunogenicity and cross-protection of the designed LAIV+4M2e vaccines, they still need further improvement. As previously shown by several groups, match between sequence of M2e in M2e-based vaccine and sequence of M2e from the challenge influenza viruses is crucial [63,64,65]. Our recent phylogenetic analysis of all M2e sequences revealed the presence of 6 host-related lineages of M2e, and we suggested 4 different consensus sequences for future M2e-based vaccine design [9]. In the current study, challenge viruses had a high level of similarity with the consensus 4M2e-epitopes (Table S1). M2e fragment of S.A. H1N1pdm09 and Cal MA H1N1pdm09 differed from avian M2e (aM2e) sequence in the 16th residue only, which is unlikely for having a role in recognition of challenge viruses by anti-M2e-antibodies. Nevertheless, a side-by-side comparison of the M2e-based vaccines with slightly different consensus sequences would shed light on the importance of these minor sequence mismatches for the cross-protective activity of the universal vaccine prototypes.

## 5. Conclusions

Here, we constructed a new M2e-based universal influenza vaccine candidate based on pre-pandemic H7N9 live attenuated influenza vaccine backbone. Given the proved safety and immunogenicity in humans of this viral vector platform, as well as the ability of the designed vaccine prototype to induce cross-protective M2e-specific antibodies in a mouse model, the new construct can be further moved for full pre-clinical characterization, including safety and immunogenicity studies in a ferret model, and a phase 1 clinical trial.

## Figures and Tables

**Table 1:** Infectivity and replication of chimeric H7N9+4M2e and its H7N9 counterpart in mice.

Virus	MID_50_, log_10_ EID_50_		Infectious Virus Titer 3 dpi at Indicated Dose			
			7.0 log_10_ EID_50_		6.0 log_10_ EID_50_	
	Nasal Turbinate	Lung	Nasal Turbinate	Lung	Nasal Turbinate	Lung
H7N9	4.5	≥6	3.6 ± 1.1	1.6 ± 0.3	2.7 ± 1.8	2.1 ± 0.5
H7N9+4M2e	4	≥6	3.7 ± 0.5	1.3 ± 0.2	3.1 ± 0.6	1.4 ± 0.3

## Data Availability

The data presented in this study are available on request from the corresponding author.

## Supplementary Materials

The following are available online at https://www.mdpi.com/2227-9059/9/2/133/s1, Figure S1: Gating strategy for NK cell degranulation assay. Figure S2: Serum IgG (A); IgG1 (B); and IgG2a (C) antibody responses in BALB/c mice immunized with H7N9+4M2e and H7N9 LAIVs six days after challenge with A/South Africa/3626/2013 (H1N1pdm09), Table S1: Sequences of M2e-epitopes of influenza viruses used in this study.
