# Supplementary material for: A Strategy to Elicit M2e-Specific Antibodies Using a Recombinant H7N9 Live Attenuated Influenza Vaccine Expressing Multiple M2e Tandem Repeats
Source: Biomedicines. 2021 Feb 1;9(2):133. doi: 10.3390/biomedicines9020133 (PMC7912525; doi:10.3390/biomedicines9020133)
Supplement: Supplementary file 1 [file biomedicines-09-00133-s001.pdf]

Supplement Materials for manuscript "A strategy to elicit M2e-specific antibodies using a recombinant H7N9 live attenuated influenza vaccine expressing multiple M2e tandem repeats"

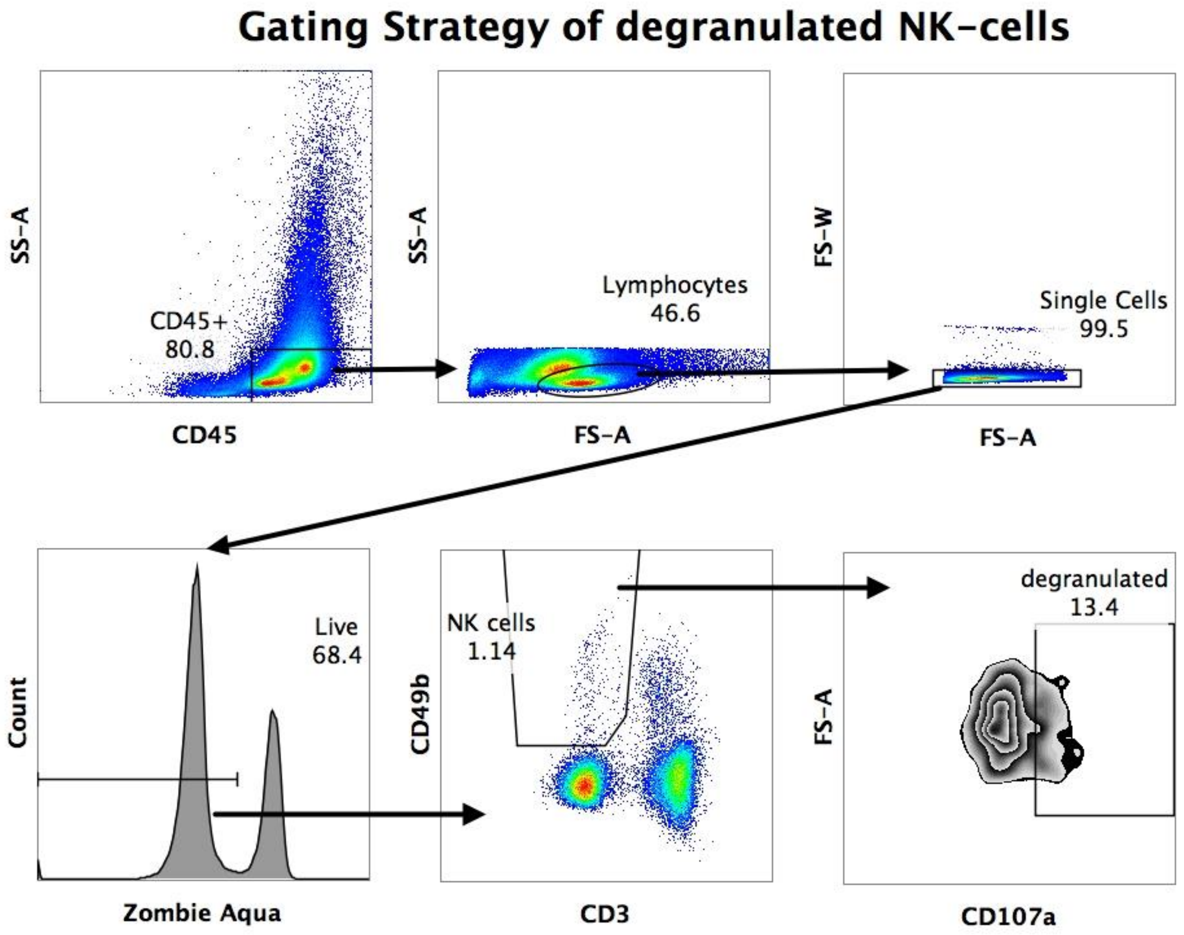

Figure S1. Gating strategy for NK cell degranulation assay.

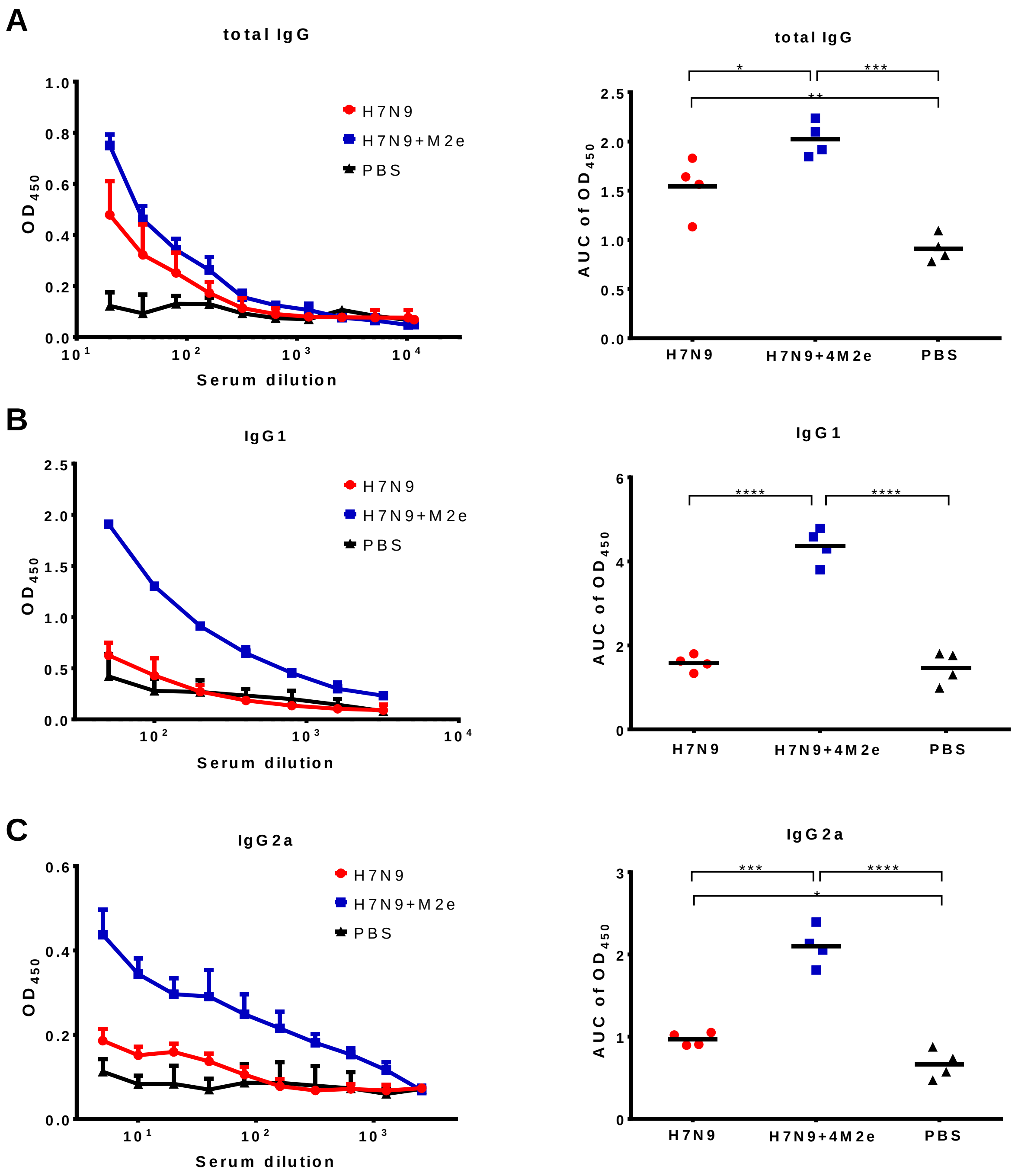

Figure S2. Serum IgG (A), IgG1 (B) and IgG2a (C) antibody responses in BALB/c mice immunized with H7N9+4M2e and H7N9 LAIVs six days after challenge with A/South Africa/3626/2013 (H1N1pdm09). Left panel shows the mean+SD of OD_{450} values; right panel shows the area under the OD_{450} curve (AUC) values for individual animal. The AUC data were analyzed with one-way ANOVA followed by a Tukey's multiple comparison test (* - $p < 0.05$; ** - $p < 0.01$; *** - $p < 0.001$; **** - $p < 0.0001$).

Table S1. Sequences of M2e-epitopes of influenza A viruses used in this study.

	1	2	3	4	5	6	7	8	9	10	11	12	13	14	15	16	17	18	19	20	21	22	23	24
human	M	S	L	L	T	E	V	E	T	P	I	R	N	E	W	G	S	R	S	N	D	S	S	D
avian	M	S	L	L	T	E	V	E	T	P	T	R	S	E	W	G	S	R	S	S	D	S	S	D
swine	M	S	L	L	T	E	V	E	T	P	T	R	N	E	W	E	S	R	S	S	D	S	S	D
H3N2	M	S	L	L	T	E	V	E	T	P	I	R	N	E	W	G	C	R	C	N	D	S	S	D
H9N2	M	S	L	L	T	E	V	E	T	L	T	R	N	G	W	E	C	K	C	S	D	S	S	D
PR8	M	S	L	L	T	E	V	E	T	P	I	R	N	E	W	G	C	R	C	N	G	S	S	D
H1N1 SA	M	S	L	L	T	E	V	E	T	P	T	R	S	E	W	E	C	R	C	S	G	S	S	D
